# Estimating the Net Contribution of Interleukin-28B Variation to Spontaneous Hepatitis C Virus Clearance

**DOI:** 10.1002/hep.24263

**Published:** 2011-05

**Authors:** Julia di Iulio, Angela Ciuffi, Karen Fitzmaurice, Dermot Kelleher, Margalida Rotger, Jacques Fellay, Raquel Martinez, Sara Pulit, Hansjakob Furrer, Huldrych F Günthard, Manuel Battegay, Enos Bernasconi, Patrick Schmid, Bernard Hirschel, Eleanor Barnes, Paul Klenerman, Amalio Telenti, Andri Rauch, and the Swiss HIV Cohort Study

**Affiliations:** 1 Institute of Microbiology, University Hospital Center, University of LausanneLausanne, Switzerland; 2 Oxford National Institute for Health Research Biomedical Research Centre and Nuffield Department of Clinical Medicine, Oxford UniversityOxford, United Kingdom; 3 Institute of Molecular Medicine, University of DublinDublin, Ireland; 4 Brigham and Women's Hospital, Harvard Medical SchoolBoston, MA; 5 University Clinic of Infectious Diseases, University Hospital Bern, University of BernBern, Switzerland; 6 Division of Infectious Diseases and Hospital Epidemiology, University Hospital Zurich, University of ZurichZurich, Switzerland; 7 Infectious Diseases and Infection Control Clinic, Department of Medicine, University Hospital BaselBasel, Switzerland; 8 Infectious Diseases Service, Regional HospitalLugano, Switzerland; 9 Division of Infectious Diseases, Canton HospitalSt. Gallen, Switzerland; 10 Division of Infectious Diseases, University HospitalGeneva, Switzerland

## Abstract

The identification of associations between interleukin-28B (*IL-28B*) variants and the spontaneous clearance of hepatitis C virus (HCV) raises the issues of causality and the net contribution of host genetics to the trait. To estimate more precisely the net effect of *IL-28B* genetic variation on HCV clearance, we optimized genotyping and compared the host contributions in multiple- and single-source cohorts to control for viral and demographic effects. The analysis included individuals with chronic or spontaneously cleared HCV infections from a multiple-source cohort (n = 389) and a single-source cohort (n = 71). We performed detailed genotyping in the coding region of *IL-28B* and searched for copy number variations to identify the genetic variant or haplotype carrying the strongest association with viral clearance. This analysis was used to compare the effects of *IL-28B* variation in the two cohorts. Haplotypes characterized by carriage of the major alleles at *IL-28B* single-nucleotide polymorphisms (SNPs) were highly overrepresented in individuals with spontaneous clearance versus those with chronic HCV infections (66.1% versus 38.6%, *P* = 6 × 10^−9^). The odds ratios for clearance were 2.1 [95% confidence interval (CI) = 1.6-3.0] and 3.9 (95% CI = 1.5-10.2) in the multiple- and single-source cohorts, respectively. Protective haplotypes were in perfect linkage (*r*^2^ = 1.0) with a nonsynonymous coding variant (rs8103142). Copy number variants were not detected. *Conclusion:* We identified *IL-28B* haplotypes highly predictive of spontaneous HCV clearance. The high linkage disequilibrium between *IL-28B* SNPs indicates that association studies need to be complemented by functional experiments to identify single causal variants. The point estimate for the genetic effect was higher in the single-source cohort, which was used to effectively control for viral diversity, sex, and coinfections and, therefore, offered a precise estimate of the net host genetic contribution. (Hepatology 2011;53:1446-1454)

Hepatitis C virus (HCV) infections resolve spontaneously in 30% to 50% of cases.^1^ Sex, ethnicity, jaundice, and coinfections with human immunodeficiency virus (HIV) and hepatitis B virus affect spontaneous HCV clearance.^2^ Recently, genetic variation in the interleukin-28B gene (*IL-28B*) was shown to strongly predict viral clearance,^3-5^ with similar effects found in HCV-monoinfected and HIV/HCV-coinfected individuals.^3^,^4^ The two strongest genetic predictors for spontaneous HCV clearance, the rs12979860 C allele and the rs8099917 T allele, are located 3 and 8 kb upstream of the *IL-28B* gene, respectively.^3^ However, at this stage, it is uncertain whether these polymorphisms play a causal role or are merely tagging one or more unknown causal variants. To identify the functional variant tagged by the associated single-nucleotide polymorphism (SNP), we performed comprehensive genetic mapping of the region to identify the most plausible causal SNP for spontaneous HCV clearance.

HCV-specific immune responses influence clinical outcomes and strongly depend on the viral genotype.^2^,^6^ Ideally, the identification of the optimal host genetic marker for spontaneous HCV clearance would control for this viral variable. However, because the genotype of the infecting virus is unknown in most infected individuals once they have cleared the virus, an assessment of the relative contribution of host genetics versus viral genetics to HCV clearance is rarely possible. Single-source outbreaks provide a unique opportunity for studying the net host genetic effects independently of the viral genotype and diversity. Therefore, we assessed the influence of *IL-28B* variants on spontaneous HCV clearance in a cohort of women infected by an identical HCV genotype 1b strain through contaminated anti-D immunoglobulins, and we compared the genetic effects in this single-source source and a multiple-source cohort.

The aim of this study was to improve the prediction of spontaneous HCV clearance through (1) the optimization of *IL-28B* genotyping and (2) the estimation of the net host genetic effects with a single-source outbreak.

## Patients and Methods

### Study Population

The multiple-source cohort included 389 randomly selected HIV/HCV-coinfected individuals (200 with spontaneous HCV clearance and 189 with chronic hepatitis C). The study participants were recruited through the Swiss HIV Cohort Study. The cohort included HIV-infected individuals coinfected with diverse HCV genotypes and was heterogeneous with respect to sex, viral coinfections, and age (Supporting Table 1).

**Table 1:** Association Between the *IL-28B* Genotype and HCV Clearance (Multiple-Source Cohort)

		Minor Allele Frequency			
				
	SNP	HCV Clearance	HCV Chronicity	Odds Ratio for Spontaneous Clearance (Noncarrier Versus Carrier)	*P* Value*
Tagging SNPs	rs8099917	0.12	0.23	2.5 (1.6-3.9)	9 × 10^−5^
	rs12979860	0.20	0.36	3.0 (1.9-4.5)	5 × 10^−7^
Candidate causal SNPs	rs4803219	0.19	0.32	2.6 (1.7-3.8)	1 × 10^−5^
	rs28416813	0.20	0.35	3.0 (1.9-4.5)	5 × 10^−7^
	rs8103142	0.21	0.36	3.0 (2.0-4.6)	2 × 10^−7^
	rs4803217	0.20	0.35	3.0 (2.0-4.6)	3 × 10^−7^

* *P* values were adjusted for sex and coinfection with hepatitis B.

Individuals from the single-source outbreak were part of a cohort of 704 women infected with HCV genotype 1b through contaminated anti-D immunoglobulins as described elsewhere.^7^ Infections occurred between May 1977 and November 1978 in Ireland. From this cohort, we included those currently attending routine clinical follow-up; 27 experienced spontaneous HCV clearance, and 44 had chronic hepatitis C. All study participants gave informed consent, which included genetic testing.

*Spontaneous HCV clearance* was defined as HCV seropositivity (determined with an enzyme-linked immunosorbent assay and confirmed by immunoblotting) and negative HCV RNA findings by quantitative or qualitative assays more than 12 months after HCV seropositivity. *Chronic hepatitis C* was defined as HCV seropositivity and detectable HCV RNA more than 12 months after HCV seropositivity. Demographic characteristics are shown in Supporting Table 1.

### Genotyping and Copy Number Variation (CNV) Determination

Because our previous genome-wide association study clearly indicated that the association signal maps to *IL-28B*, we further explored this gene in more detail. On the basis of our previous work on the resequencing of the *IL-28B* locus,^3^ the genotyping of four candidate causal SNPs (rs4803219, rs28416813, rs8103142, and rs4803217) was performed by TaqMan allelic discrimination (ABI-Prism 7000 SDS software, Applied Biosystems) with custom Assays-on-Demand products from Applied Biosystems; this was preceded by a pre-amplification step. Primers and probes are shown in Supporting Table 2. In addition, the tag SNP rs12979860 was assessed with a custom TaqMan assay designed by Ge et al.,^8^ and the tag SNP rs8099917 was genotyped with an Assays-on-Demand product provided by Applied Biosystems (C__11710096_10). Haplotype inference was performed with Phase 2.1 software (University of Washington, Seattle, WA).

An analysis of CNVs (deletions, insertions, and inversions) was initiated through an inspection of the structural variation at the *IL-28B* locus and 1 Mb (1 million nucleotides) upstream and downstream of the gene in chromosome 19 (as captured in the 1000 Genomes Project; go to http://www.1000genomes.org for the March 2010 data release).^9^ The analysis included calls from both pilot 1 [179 samples sequenced with low-pass shotgun (2-6×) coverage] and pilot 2 [6 individuals with high (>30×) coverage] of the 1000 Genomes Project. CNV calls from the 1000 Genomes Project are reported as validated, unvalidated, and invalidated on the basis of a number of algorithms, experimental data, and replication across various samples and populations.

The presence of CNVs was then experimentally assessed by quantitative real-time polymerase chain reaction (PCR); the primers and probes are shown in Supporting Table 2. The CNV assessment with real-time PCR included a clone containing one copy of *IL-28B* and the housekeeping gene hydroxymethylbilane synthase as a control (Supporting Table 2). Because the quantitative PCR approach allowed the detection of CNVs only in a limited region of the gene (set by the location of the chosen primers), we further assessed potential CNVs with primer combinations that would amplify a PCR product only in the presence of CNVs (Supporting Fig. 1). The targeted PCR across potential CNVs was internally controlled by the identification of the expected amplification fragment or larger/shorter fragments.

### Statistics

Genetic associations were assessed by logistic regression. The analyses were adjusted for covariates significantly associated with spontaneous HCV clearance in univariate analyses. Haplotype analyses were performed with the haplologit command in the Stata software package (version 10.0). This analysis included haplotypes exceeding a frequency of 2/N (the default threshold). The estimates of haplotype effects were adjusted for significant covariates in logistic regression models.

## Results

### Genotyping Candidate Causal Variants

**Fig. 1:**
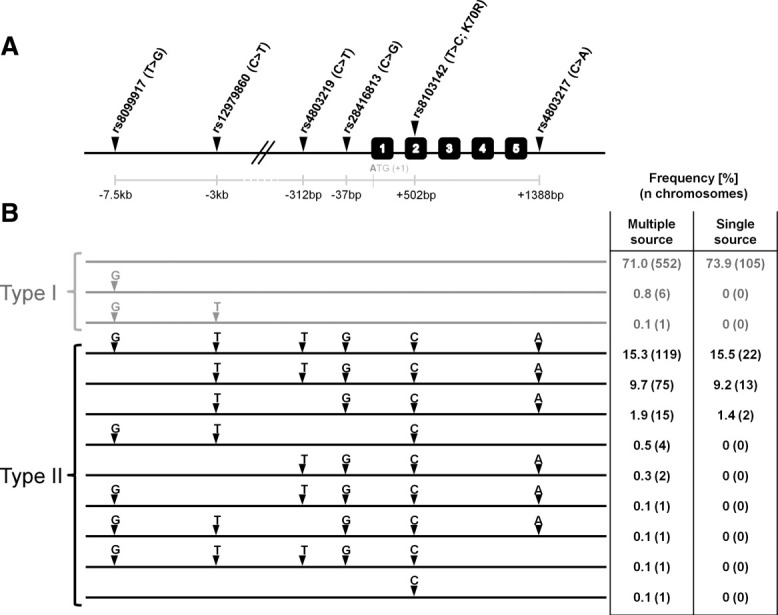
*IL-28B* genotyping and haplotype inference. (A) Two tagging SNPs (rs8099917 and rs12979860) and four candidate causal SNPs in *IL-28B* were genotyped in all study participants. Boxes 1 to 5 denote exons. (B) Haplotype inference identified 12 distinct haplotypes divided into two main families (types I and II). Arrowheads indicate the presence of the minor allele.

Our previous genome-wide association study identified rs8099917 as the strongest predictor of spontaneous HCV clearance.^3^ This variant is located within a genomic block encompassing the *IL-28B* gene and is, therefore, susceptible to reflecting the signal resulting from a functional variant in *IL-28B*. To identify the potentially causal variant tagged by rs8099917, *IL-28B* had been previously resequenced in 41 HIV/HCV-coinfected individuals with the four extreme genotype/phenotype combinations: chronic or spontaneously cleared hepatitis C and homozygosity for the protective or risk alleles.^3^ Candidate causal SNP selection was based on location, frequency among the main haplotype families, and linkage disequilibrium.^3^ This approach led to the prioritization of four SNPs as prime candidates for causality (Fig. 1A). These four SNPs were genotyped along with the two tagging SNPs that were most strongly associated with spontaneous HCV clearance in previous studies (rs8099917 and rs12979860^3^,^4^). Twelve distinct haplotypes could then be derived from the six genotyped SNPs, as shown in Fig. 1B. The haplotypes were further categorized according to the classification used in our previous study into two main families (Fig. 1B). Type I haplotypes were characterized by the presence of the common allele for all candidate SNPs (Fig. 1B).

### Genotyping Assay Optimization

**Fig. 2:**
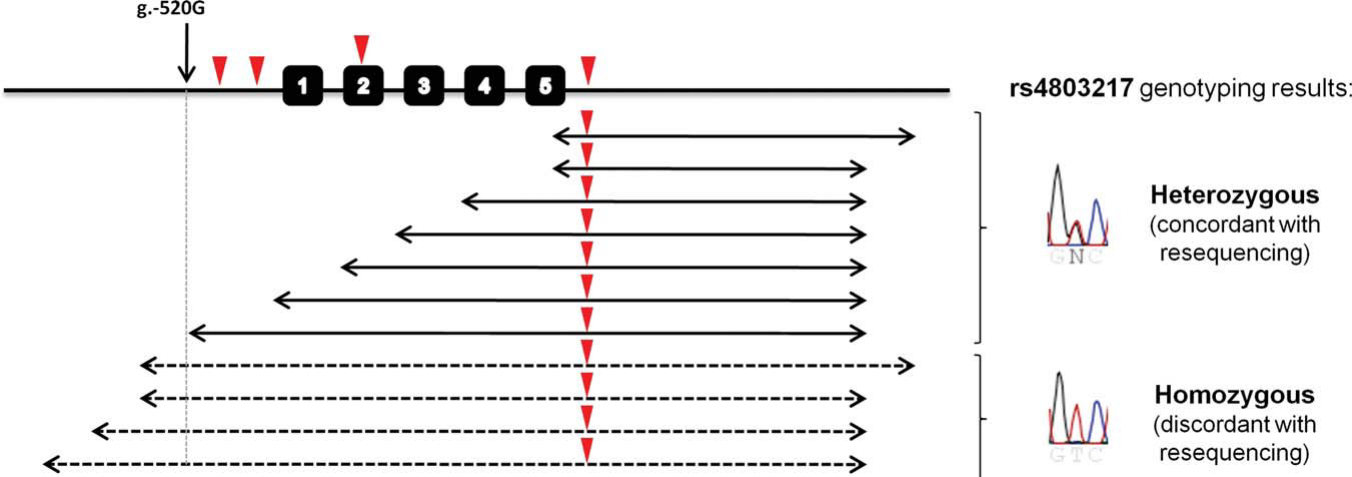
Primer pair optimization for the pre-amplification step. Because discordant results were obtained by genotyping and resequencing, several primer combinations were tested for the pre-amplification step preceding the genotyping. The arrows show the different PCR products amplified for the pre-amplification step. Arrows with dashed lines indicate discordant results for a given SNP (heterozygous by resequencing and homozygous by the TaqMan assay), whereas arrows with solid lines indicate concordant results for a given SNP (heterozygous by both resequencing and TaqMan assay). A primer combination containing a forward primer located upstream of position g.-520G yielded discordant results in up to 19.5% of the individuals (for rs8103142). The red triangles show the location of the four genotyped SNPs.

Because of the high homology between the *IL-28B* and *IL-28A* sequences (96% amino acid identity^10^), we first performed a pre-amplification step in order to selectively genotype *IL-28B* polymorphisms in the TaqMan assays. The four candidate SNPs were then genotyped from the pre-amplified product. However, we observed discrepancies in up to 19.5% of cases for rs8103142 between the TaqMan results and the previous resequencing results.^3^ Discrepancies were present for various pre-amplification primer combinations (marked with dashed lines in Fig. 2), with more frequent homozygosity found with the TaqMan assay; this suggested the preferential amplification of only one allele in some individuals. Further optimization of the pre-amplification step identified primer combinations that correctly identified all genotypes (marked with solid lines in Fig. 2). Primer combinations that included a forward primer downstream of position g.-520G gave concordant results. Primers and probes that were used in the optimized genotyping assay are listed in Supporting Table 2.

### CNVs

**Fig. 3:**
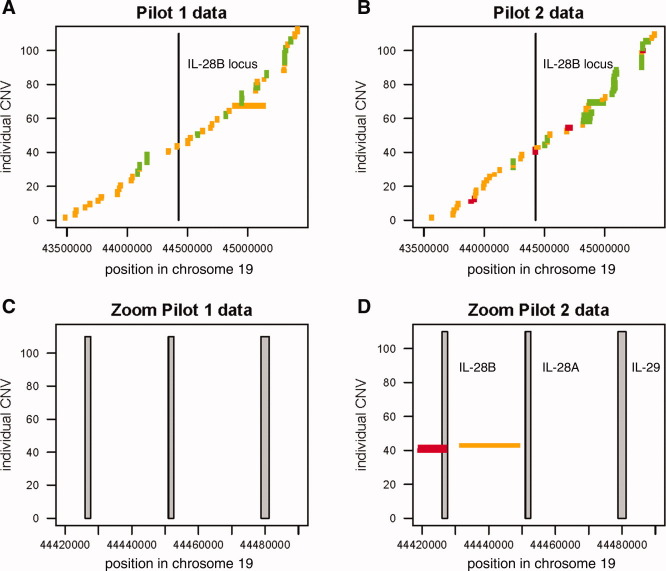
CNVs at the *IL-28B* locus. (A,B) CNV data from the 1000 Genomes Project (pilots 1 and 2) for 1 Mb upstream and downstream of the *IL-28B* locus (black, vertical lines) in chromosome 19. The *y* axis and the horizontal color lines represent individual observations of possible CNVs (orange, not yet confirmed; red, refuted/invalidated; green, confirmed/validated). (C,D) Close-ups of the locus. The gray, vertical lines indicate the chromosomal locations of *IL-28B*, *IL-28A*, and *IL-29*.

To further characterize the genetic region, we assessed whether CNVs (deletions, insertions, and inversions) exist in the *IL-28B* region. An analysis of pilot 1 data from the 1000 Genomes Project identified 113 possible instances of CNVs in the window of 2 Mb around the *IL-28B* locus (Fig. 3A). These correspond to 107 deletions and 6 insertions; 43 of these have been validated, and validation is pending for 70. An analysis of the pilot 2 data identified 110 possible instances of CNVs; these correspond to 106 deletions, 3 duplications, and 1 inversion, and 48 have been validated. Validation is pending for 53, and 9 have been invalidated (Fig. 3B). However, none of the reported CNVs directly involve *IL-28B*, with the exception of an inversion (yet to be validated) reported in one individual in the *IL-28B*–*IL-28A* intergenic region (Fig. 3C,D).

We could not identify any CNV with quantitative real-time PCR using primers amplifying a region of 121 bp that spanned exon 5 and the 3′-untranslated region (data not shown). The customized PCR described in Supporting Fig. 1A revealed potential gene duplications; however, these were entirely explained by artifactual formation of gene copies due to PCR-induced recombination of repetitive regions in the *IL-28B* region (Supporting Fig. 1B).^11^

### Predictive Value of Candidate SNPs for Spontaneous HCV Clearance in the Multiple-Source Cohort

**Fig. 4:**
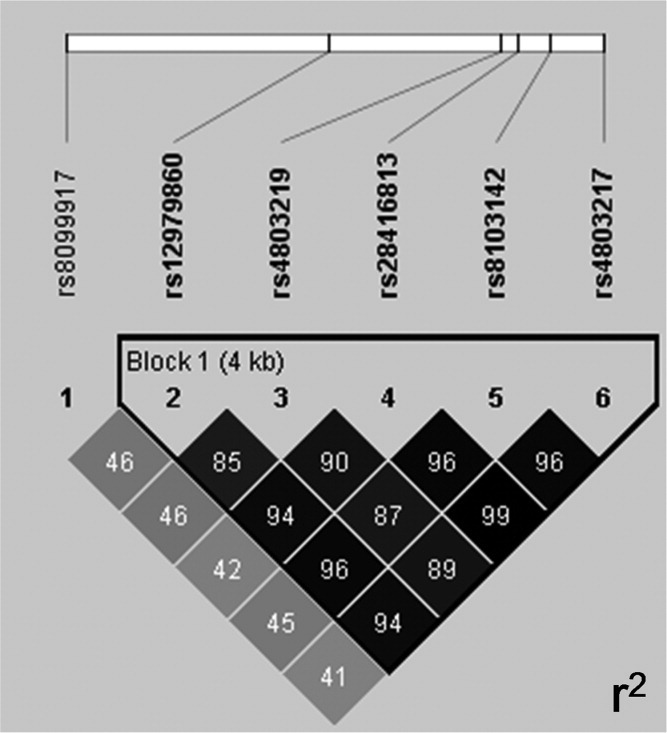
Linkage disequilibrium plot for the multiple-source cohort. The linkage disequilibrium between the four candidate causal SNPs (rs4803219, rs28416813, rs8103142, and rs4803217) and the two previously identified tagging SNPs is shown.

Using the optimized pre-amplification step, we genotyped the four candidate causal variants in the entire cohort. All SNPs were highly associated with spontaneous HCV clearance and had a stronger effect in comparison with the tagging SNP rs8099917 identified in our previous genome-wide association study^3^ (Table 1). Using nested regression models, we observed that rs8099917 was not significantly associated after an adjustment for candidate SNPs (*P* > 0.1 for all comparisons), whereas the candidate causal variants remained significantly associated with spontaneous HCV clearance after we accounted for rs8099917 (*P* < 0.001 for all comparisons). The linkage disequilibrium between rs8099917 and the candidate causal variants was moderate (*r*^2^ ∼ 0.4; Fig. 4), whereas the other tag SNP, rs12979860,^8^ was highly linked to the candidate variants (*r*^2^ ≥ 0.85, Fig. 4).

### Predictive Value of Candidate SNPs for Spontaneous HCV Clearance in the Single-Source Cohort

We next investigated the net host genetic contribution to spontaneous HCV clearance in the single-source cohort. Again, all genotyped SNPs were significantly associated with spontaneous HCV clearance. The candidate causal SNPs were more strongly associated than the tagging SNP rs8099917 and were in perfect linkage disequilibrium with rs12979860, except for rs4803219 (*r*^2^ = 0.93; Table 2).

**Table 2:** Association Between the *IL-28B* Genotype and HCV Clearance (Single-Source Cohort)

		Minor Allele Frequency			
				
	SNP	HCV Clearance	HCV Chronicity	Odds Ratio for Spontaneous Clearance (Noncarrier Versus Carrier)	*P* Value*
Tagging SNPs	rs8099917	0.08	0.20	3.3 (1.0-11.2)	0.05
	rs12979860†	0.13	0.34	4.1 (1.4-11.8)	0.008
Candidate causal SNPs	rs4803219	0.11	0.33	4.6 (1.6-13.6)	0.005
	rs28416813†	0.13	0.34	4.1 (1.4-11.8)	0.008
	rs8103142†	0.13	0.34	4.1 (1.4-11.8)	0.008
	rs4803217†	0.13	0.34	4.1 (1.4-11.8)	0.008

* *P* values were adjusted for sex and coinfection with hepatitis B.

† In perfect linkage disequilibrium in this cohort.

### IL-28B Haplotypes and Spontaneous HCV Clearance

**Fig. 5:**
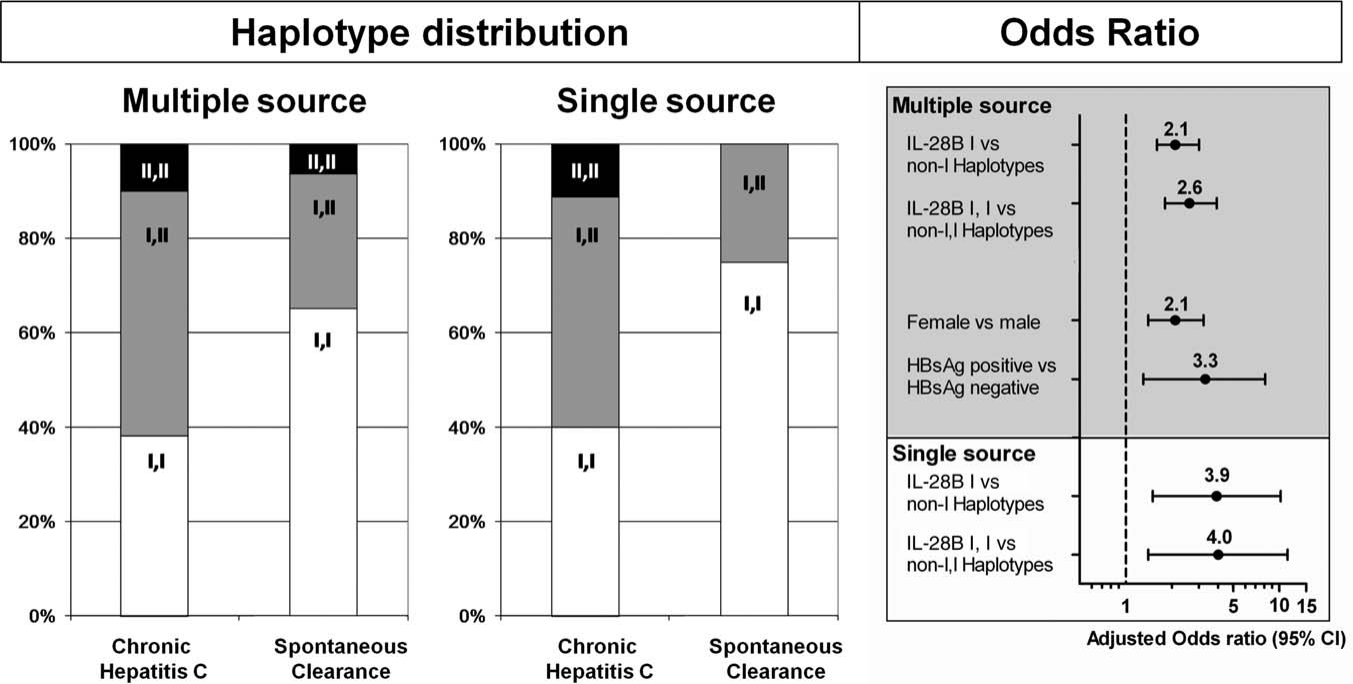
*IL-28B* haplotypes and spontaneous HCV clearance in the multiple- and single-source cohorts. The odds ratios were adjusted for *IL-28B* haplotypes, sex, and coinfection with hepatitis B. Abbreviation: HBsAg, hepatitis B surface antigen.

We next examined the association of *IL-28B* haplotypes with spontaneous HCV clearance. In both cohorts, haplotypes characterized by carriage of the major alleles at *IL-28B* candidate SNPs had the strongest association with clearance (Table 3). These type I haplotypes (Fig. 1B) were clearly enriched in individuals with spontaneous HCV clearance, and there was a greater association in the single-source cohort (Fig. 5). Homozygosity for type I haplotypes was highly linked to the rs12979860 CC genotype (*r*^2^ = 0.97).

**Table 3:** Association Between the *IL-28B* Haplotypes and HCV Clearance

*IL-28B* SNP in the Promoter/Coding Region and 3′-Untranslated Region*				Multiple-Source Cohort		Single-Source Cohort	
		
rs4803219 (C>T)	rs28416813 (C>G)	rs8103142 (T>C)	rs4803217 (C>A)	Odds Ratio (95% CI)†	*P* Value	Odds Ratio (95% CI)†	*P* Value
0	0	0	0	2.1 (1.6-3.0)	2 × 10^−6^	3.9 (1.5-10.2)	0.005
0	0	1	0	1.4 (0.2-8.6)	0.7	—‡	—‡
0	1	1	1	0.3 (0.1-0.9)	0.04	—‡	—‡
1	1	1	1	0.5 (0.3-0.7)	2 × 10^−5^	0.3 (0.09-0.7)	0.006

* 0 = major allele; 1 = minor allele.

† Odds ratios were adjusted for *IL-28B* haplotypes, sex, and coinfection with hepatitis B.

‡ The numbers were too small for odds ratio calculations.

## Discussion

Genetic variation in *IL-28B* is the strongest known genetic predictor of natural and treatment-induced clearance of HCV infection. Although there is overwhelming evidence for the association of SNPs with HCV infection outcomes, the causal genetic variant has not been identified yet. Using a comprehensive and optimized genetic analysis of *IL-28B*, we evaluated SNPs in the coding, promoter, and 3′-untranslated regions of the gene that could represent causal variants. Haplotypes characterized by carriage of the major alleles at the *IL-28B* candidate SNPs (type I haplotypes) were highly overrepresented in individuals with spontaneous HCV clearance versus those with chronic HCV infections. Homozygosity for the rs12979860 C allele (CC genotype) tagged (*r*^2^ = 0.97) homozygosity for the protective type I haplotypes. Protective type I haplotypes were in perfect linkage (*r*^2^ = 1.0) with a nonsynonymous coding variant (rs8103142) that has also been highly associated with treatment response.^8^,^12^ Although rs8099917 was only moderately linked to the SNPs in the *IL-28B* gene, there was a high linkage of rs12979860 with the four candidate causal SNPs in both cohorts. The candidate causal SNPs and haplotypes were, therefore, better tagged by rs12979860 than rs8099917. Furthermore, rs12979860 and the candidate causal SNPs remained significantly associated with HCV clearance after we accounted for rs8099917. In contrast, rs8099917 was not significantly associated after an adjustment for candidate causal SNPs. Therefore, we conclude that candidate causal SNPs and rs12979860 are better predictors of spontaneous HCV clearance than rs8099917. The candidate SNPs are in very high linkage disequilibrium (Fig. 4). This indicates that association studies are not sufficient to identify the causal SNPs, at least in Caucasians. Functional studies are needed to define which variants ultimately influence the control of HCV infection.

Even if protective *IL-28B* genotypes were clearly enriched in individuals with spontaneous HCV clearance in both cohorts, the point estimate of the odds ratio was approximately 2-fold higher in the single-source cohort. Women homozygous for the protective type I haplotypes were approximately 4 times more likely to spontaneously clear their HCV infection in comparison with women carrying at least one type II haplotype. Because of the inherent control of clinical, demographic, and viral factors in the single-source cohort, this point estimate represents an optimal assessment of the net host genetic contribution. This finding is in line with a very recent report by Tillmann and colleagues,^13^ who found a strong association between the rs12979860 CC genotype and spontaneous HCV clearance in a German single-source cohort. In the multiple-source cohort, the effects of *IL-28B* haplotypes on spontaneous HCV clearance were similar in men [odds ratio = 2.3, 95% confidence interval (CI) = 1.5-4.2] and women (odds ratio = 2.3, 95% CI = 1.5-3.6). Furthermore, *IL-28B* has very similar effects on spontaneous HCV clearance in HIV/HCV-coinfected and HCV-monoinfected individuals.^3-5^ Although the inherent heterogeneity between the two cohorts makes it difficult to compare predictors of spontaneous HCV clearance, it is unlikely that the larger effect in the single-source cohort can be explained entirely by the different distributions of sex and coinfection with HIV. The influence of viral genotypes on spontaneous HCV clearance rates is poorly understood. This is mainly due to the paucity of acute hepatitis C cohorts in which the viral genotype can be assessed in individuals who spontaneously clear their infections. The limited overlap of T cell responses between HCV genotypes^6^ suggests that the viral genotype influences the host immune response to HCV. It is, therefore, not surprising that the effect of *IL-28B* variants was larger in the single-source cohort, which was used to control for viral effects. Although the difference in the odds ratios between the single- and multiple-source cohorts was not statistically significant with overlapping CIs, it is interesting to note that the highest odds ratios for spontaneous HCV clearance were observed in the Irish and German single-source cohorts. This underscores the advantages of single-source cohorts for genetic association studies of infectious diseases because these cohorts allow the net host genetic determinants to be assessed independently of pathogen-associated variability.

Our study also identified substantial technical complexity in the genetic analysis of the *IL-28B* locus. First, the high homology between *IL-28A* and *IL-28B* requires pre-amplification of *IL-28B* before the genotyping of SNPs in the promoter, coding, and 3′-untranslated regions of *IL-28B* by TaqMan in order to avoid unspecific amplification of *IL-28A*, which can result in erroneous genotypes. Second, the pre-amplification step needs optimized primer combinations; upstream of position g.-520G, the forward primer does not reliably amplify both alleles, and this can result in erroneous genotype distributions for SNPs in the promoter, coding, and 3′-untranslated regions of *IL-28B*. Tagging SNPs can be genotyped reliably without a pre-amplification step because these are not located in a region with high homology with *IL-28A*. Therefore, our findings do not alter the conclusions of previous reports on the impact of the tagging SNPs rs12979860 and rs8099917 on the control of HCV infection. Third, repetitive homology regions can artifactually simulate CNVs. The study also proceeded to an analysis of CNVs, which were a consideration because of the close proximity of the *IL-28B* and *IL-28A* paralogs. The analysis of CNVs captured in the 1000 Genomes Project identified several validated CNVs near *IL-28B*; however, none of these encompassed the *IL-28B* gene. Furthermore, neither real-time nor targeted PCR identified CNVs. It is, therefore, unlikely that CNVs play a role in the strong influence of *IL-28B* variation on HCV infection outcomes. Completion of the 1000 Genomes Project, which will yield many more samples sequenced with high coverage, may lead to a better understanding of the genetic structure around the *IL-28B* locus.

Our study has confirmed and extended previous observations. We have confirmed the high linkage of rs12979860 to candidate causal *IL-28B* SNPs observed in the Individualized Dosing Efficacy Versus Flat Dosing to Assess Optimal Pegylated Interferon Therapy (IDEAL) cohort,^8^ and we have additionally described the influence of these variants on spontaneous HCV clearance. Furthermore, we have demonstrated that the rs12979860 CC genotype tags homozygosity for protective type I haplotypes that are characterized by carriage of major alleles of *IL-28B* candidate causal SNPs. We have also described the substantial complexity of the genetic analysis of the *IL-28B* locus, and we have presented a new approach to identifying samples with particular *IL-28B* genotypes for functional studies. Our study has also confirmed the strong effect of *IL-28B* variation in the German single-source cohort,^13^ has extended the genetic assessment, and has provided comparative estimates of multiple- and single-source cohorts and thereby effectively defined the net host contribution to the phenotype. Finally, previous studies that assessed the potential role of CNVs in HCV infection outcomes^8^ relied on the information provided by HapMap (see http://hapmap.ncbi.nlm.nih.gov). Because this approach provides relatively poor discrimination for detecting CNVs, we performed an extensive screening of the region with the 1000 Genomes Project data and real-time and targeted PCR. These studies and the current work jointly represent a definitive assessment of genetic variation at the *IL-28B* locus and indicate the limits of genetic approaches to the understanding of the mechanism of action of *IL-28B*.

In conclusion, SNPs located in the promoter, coding, and 3′-untranslated regions of *IL-28B* are highly associated with spontaneous HCV clearance. These SNPs are prime candidates for functional assessment. The method described here can be useful to identify samples with particular *IL-28B* genotypes for functional studies. We also have confirmed that for large-scale studies, rs12979860 genotyping is a reliable method for predicting the presence or absence of the putative causal haplotypes in *IL-28B* associated with spontaneous HCV clearance. The striking association of *IL-28B* genotypes with spontaneous HCV clearance in a single-source cohort underscores the role of host genetic variation in the control of HCV infection, which is independent of viral effects.

## Abbreviations

CI: confidence interval
CNV: copy number variation
HBsAg: hepatitis B surface antigen
HCV: hepatitis C virus
HIV: human immunodeficiency virus
IL: interleukin
PCR: polymerase chain reaction
SNP: single-nucleotide polymorphism
