# Supplementary material for: Estimating the Net Contribution of Interleukin-28B Variation to Spontaneous Hepatitis C Virus Clearance
Source: Hepatology. 2011 May;53(5):1446–54. doi: 10.1002/hep.24263 (PMC3128709; doi:10.1002/hep.24263)
Supplement: Supplementary file 1 [file hep0053-1446-SD1.doc]

Supporting Table 1. Demographics

|  | **Multiple source cohort^$^** | | **Single Source Cohort**^#^ | |
| --- | --- | --- | --- | --- |
| **Characteristics** | **Spontaneous clearance** | **Chronic infection** | **Spontaneous clearance** | **Chronic infection** |
| N | 200 | 189 | 27 | 44 |
| Age (median, IQR) | 39 (35-43) | 39 (34-42) | 29 (25-31) | 28 (26-33) |
| Female sex, n (%) | 95 (47) | 60 (31)* | 27 (100) | 44 (100) |
| HBs positive, n(%) | 20 (10) | 7 (4)** | 0 (0) | 0 (0) |
| HCV genotypes |  |  |  |  |
| 1 | unknown | 75 (40) | 28 (100) | 45 (100) |
| 2 |  | 5 (3) |  |  |
| 3 |  | 56 (30) |  |  |
| 4 |  | 21 (11) |  |  |
| Other / unknown |  | 1. (17) |  |  |

NA: Not applicable. *P=0.02, **P=0.002

^$^All HIV positive, ^#^All HIV negative

**Supporting** Table 2: Primers and Probes for genotyping.

| **Rs number** | **position** | **Primers** | **Probe** | | | **Size** | **Ref** |
| --- | --- | --- | --- | --- | --- | --- | --- |
|  |  |  |  | | |  |  |
| IL28B |  |  |  | | |  |  |
| Preamplification step | | F: 5’-GAGCAGGTGGAATCCTCTTG-3’; R: 5’- AGCAGGCACCTTGAAATGTC–3’ | | | - | 3308 bp | This study |
| rs8099917 | g.-7558A>G | Assay on demand from Applied Biosystems: C__11710096_10 | | | | unknown | AB |
| rs12979860 | g.-3180G>A | F: 5’-GCGCGGAGTGCAATTCAAC-3’; R: 5’- GCCTGTCGTGTACTGAACCA–3’ | | | VIC 5‘-TGGTTCGCGCCTTC-3‘- MGBNFQ 6FAM 5‘-CTGGTTCACGCCTTC-3‘- MGBNFQ | 66 bp | [1] |
| rs4803219 | g.-312G>A | F: 5’- AGGCTGTGTTTTCACTTTTCCTACA-3’; R: 5’- GTAATTCCTGCCTGAGCTCCAT–3’ | | | VIC 5‘-TCTGTCAGGGATAAAA-3‘- MGBNFQ 6FAM 5‘-TTCTGTCAGAGATAAAA-3‘- MGBNFQ | 88 bp | AB (custom assay) |
| rs28416813 | g.-37G>C | F: 5’- CAGCCCCTGCCCTCAG-3’; R: 5’- TGTCACAGAGAGAAAGGGAGCT–3’ | | | VIC 5‘-TGGGCAGCCTCTGCAT-3‘- MGBNFQ 6FAM 5‘-TGGGCAGCCTCTCCAT-3‘- MGBNFQ | 60 bp | AB (custom assay) |
| rs8103142 | g.502A>G | F: 5’- CTAACCTGTGCCTTTGCTGTCTA-3’; R: 5’- GCCTCAGGTCCCAGGTC–3’ | | | VIC 5‘-AGCGGCACTTGCAG-3‘- MGBNFQ 6FAM 5‘-AGCGGCACCTGCAG-3‘- MGBNFQ | 94 bp | AB (custom assay) |
| rs4803217 | g.1388G>T | F: 5’- GCCAGTCATGCAACCTGAGATTTTA-3’; R: 5’- AAATACATAAATAGCGACTGGGTGACA–3’ | | | VIC 5‘-TAGCCACTTGGCTTAAT-3‘- MGBNFQ 6FAM 5‘-TTAGCCACTTGTCTTAAT-3‘- MGBNFQ | 82 bp | AB (custom assay) |
| CNV | exon5-3’UTR | F: 5’- CCTGAATTGTGTTGCCAGC-3’; R: 5’- CATAAATAGCGACTGGGTGAC–3’ | | | 6FAM-5’- ACCCTTCCGCCAGTCATGC-3’ MGBNFQ | 121bp | This study |
|  |  |  |  | | |  |  |
| HMBS (encoding PBGD) | | | | | | | |
| **1* | CNV | F : 5’-AAGGGATTCACTCAGGCTCTTTC-3’; R : 5’-GGCATGTTCAAGCTCCTTGG-3’ | | VIC-5’-CCGGCAGATTGGAGAGAAAAGCCTGT-3’- MGBNFQ | | 75 bp | [2] |

SNP, single nucleotide polymorphism. AB, Applied Biosystems. CNV, copy number variations. Position numbering of the SNPs refers to genomic (g.) DNA (bp1=A of ATG) in the sense of the gene (opposite the sense of the genome, as *IL28B* is encoded in the negative strand). F, forward. R, reverse (according to the gene and not the genome). *HMBS*, hydroxymethylbilane synthase. PBGD, porphobilinogen deaminase.

1. Ge, D., et al., *Genetic variation in IL28B predicts hepatitis C treatment-induced viral clearance.* Nature, 2009. **461**(7262): p. 399-401.

2. Konig, R., et al., *Global analysis of host-pathogen interactions that regulate early-stage HIV-1 replication.* Cell, 2008. **135**(1): p. 49-60.

**Supporting Figure 1: *IL28B* copy number investigation and artifact.**

A*.* An approach consisting of a forward primer (in green) located downstream a reverse primer (in blue) was performed in order to identify potential gene duplication. B. The presence of repetitive and complementary region (AluJo) in *IL28B* 3’UTR resulted in the artificial formation of chimeras by template switching.
